# The Role of VHL in the Development of von Hippel-Lindau Disease and Erythrocytosis

**DOI:** 10.3390/genes13020362

**Published:** 2022-02-17

**Authors:** Petra Hudler, Mojca Urbancic

**Affiliations:** 1 Medical Centre for Molecular Biology, Institute of Biochemistry and Molecular Genetics, Faculty of Medicine, University of Ljubljana, Vrazov trg 2, 1000 Ljubljana, Slovenia; petra.hudler@mf.uni-lj.si; 2 Eye Hospital, University Medical Centre Ljubljana, Grabloviceva ulica 46, 1000 Ljubljana, Slovenia

**Keywords:** VHL, VHL disease, Chuvash polycythemia, genetic variation, erythrocytosis, pheochromocytoma, renal cell carcinoma, retinal hemangioblastoma, hemangioblastoma

## Abstract

Von Hippel-Lindau disease (VHL disease or VHL syndrome) is a familial multisystem neoplastic syndrome stemming from germline disease-associated variants of the *VHL* tumor suppressor gene on chromosome 3. VHL is involved, through the EPO-VHL-HIF signaling axis, in oxygen sensing and adaptive response to hypoxia, as well as in numerous HIF-independent pathways. The diverse roles of VHL confirm its implication in several crucial cellular processes. VHL variations have been associated with the development of VHL disease and erythrocytosis. The association between genotypes and phenotypes still remains ambiguous for the majority of mutations. It appears that there is a distinction between erythrocytosis-causing VHL variations and VHL variations causing VHL disease with tumor development. Understanding the pathogenic effects of *VHL* variants might better predict the prognosis and optimize management of the patient.

## 1. Introduction

Von Hippel-Lindau disease (VHL disease or VHL syndrome) is a familial multisystem syndrome stemming from germline disease-associated variants of the *VHL* tumor suppressor gene on chromosome 3 [1]. It is an autosomal dominant neoplastic disorder in which multiple benign and malignant tumors, as well as cysts, develop in the central nervous system (the brain, spinal cord, retina, and inner ear) and visceral organs (kidney, adrenal gland, pancreas, epididymis, and broad ligament) [2,3]. Despite its classification as a dominant disorder, the most common pattern in hereditary VHL disease is the inheritance of a germline genetic variant (herein, mutation) in one allele, followed by a second somatic change, leading to loss of the second allele [2,4,5]. In approximately 20% of cases, VHL syndrome is sporadic, caused by a de novo genetic change that arises during the formation of reproductive cells, or very early during the embryogenesis [4,6,7]. After the identification of the VHL gene in 1993, the phenotype of VHL pathogenic genetic variations was expanded to include VHL disease, dominantly inherited familial pheochromocytoma, and autosomal recessive familial polycythemia [8,9,10]. Chuvash polycythemia, a secondary congenital erythrocytosis, was the first congenital erythrocytosis linked to a VHL mutation [8]. Since then, several other VHL mutations associated with erythrocytosis have been identified [11].

The aim of this review is to provide an overview of the role of *VHL* gene mutations leading to the development of VHL disease and erythrocytosis, and to present a case of a patient with VHL disease due to a de novo mutation, to illustrate the role of genetic testing in the management of patients with genetic disease.

## 2. VHL Canonical and Non-Canonical Functions

**Figure 1:**
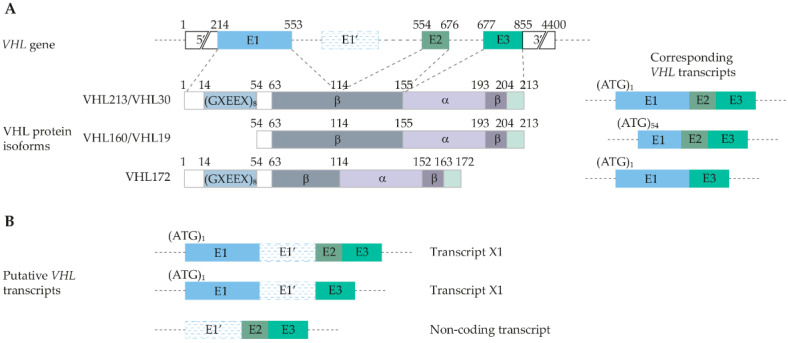
Schematic representation of *VHL* gene structure. (**A**). *VHL* gene structure and protein isoforms with corresponding *VHL* transcripts. (**B**). Putative *VHL* transcripts, containing exon E′, identified by cloning and sequencing [15].

The von Hippel-Lindau tumor suppressor gene (*VHL*) is located on chromosome 3. Initially it was thought to be composed of three exons that encode the VHL protein [12,13]. Subsequent research revealed more complex transcription patterns, and an additional cryptic exon, resulting in several different functional *VHL* isoforms (Figure 1) [14,15]. Soon after its discovery, it was confirmed that the two most commonly isolated isoforms, VHL30 or VHL213 and VHL19 or VHL160, are both biologically active and are expressed in all tissues [16,17,18].

VHL30 is the longest isoform, with a molecular mass of 30 kDa. It has 213 amino acids and consists of three exons, whereas VHL19 has 160 amino acids and a molecular mass of 19 kDa [16]. Iliopoulos et al. demonstrated that VHL19 is generated from an internal translational start at methionine 54 [16]. Another protein product of the *VHL* gene, VHL172, which arises from alternative splicing that joins exons one and three while excluding exon two, was also detected [17].

Recently, Lenglet et al. identified transcripts containing a cryptic exon, E1′, located in intron one of the *VHL* gene, spliced either with exon one or exons two and three [15]. They speculated that a theoretical protein consisting of 193 amino acids could exist, containing 114 amino acids encoded by exon one and 79 amino acids encoded by the cryptic exon, E1′. Their findings were linked to the aberrant retention of this cryptic exon in patients with VHL disease and erythrocytosis.

The reference sequence collection currently displays three mRNA and protein reference variants, which differ in length and exon structure. The three representative isoforms consist of 213, 172, and 193 amino acids residues, respectively [19]. UniProt database describes three isoforms, which are produced by alternative splicing and alternative initiation. Isoform one corresponds to RefSeq isoform one (VHL30 or VHL213) and isoform two corresponds to RefSeq isoform two (VHL172), whereas isoform three corresponds to VHL19 (160 amino acids) and is produced from alternative initiation at methionine 54 [20].

The longest protein isoform has two main protein binding domains, β and α domain, which are preceded by N-terminal acidic disordered domain [21,22]. The β domain is composed of approximately 100 amino acids. This domain is rich in β strands, which form a β-sheet structure, and interacts with hydroxylated HIFs, RNA polymerase II, protein kinase C isoforms, and other proteins [23,24]. The shorter α domain contains binding sites for Elongin-B/C (BC box), Cullin-2 (Cul-2 box), p53, and other proteins [23]. The β domain contains another binding interface, interface C, which is important for VHL localization and binds TBP1 and EEF1A [23].

On the subcellular level, the strongest VHL protein expression was observed in the cytosol, nucleus, mitochondrion and endoplasmic reticulum [25]. Interestingly, Illiopoulus et al. noted that VHL, which is composed of 213 amino acids and corresponds to VHL30, localizes predominantly to cytosol, or is membrane-associated [16]. It was found only in low levels in the nucleus, whereas the 160-amino acid VHL (VHL19) was found in both the nucleus and cytosol. In contrast to the cytosolic membrane-bound VHL30, VHL19 was not associated with cell membranes [16].

RNA sequencing of 27 different normal tissues, from 95 individuals, revealed that VHL protein is ubiquitously expressed in most tissues [26]. However, protein expression studies showed that, despite the relatively high abundance of mRNA in some tissues, the levels of VHL were low or absent, indicating that complex mechanisms govern the mRNA expression, protein synthesis, and/or protein stability of VHL (https://www.proteinatlas.org/ENSG00000134086-VHL/tissue, available from v21.0 proteinatlas.org, accessed on 10 January 2022) [26,27]. This is consistent with general observations in mammalian tissues, where a number of genes exhibited very low correlations between mRNA expression levels and protein staining (Pearson’s r ~ 0.40) [26,28].

**Figure 2:**
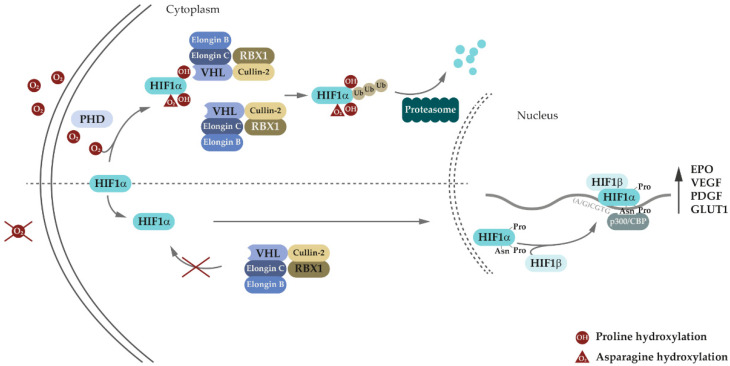
Role of VHL in adaptive response to oxygen levels.

The most important canonical role of VHL, through the EPO-VHL-HIF signaling axis, is its involvement in oxygen sensing and adaptive response to hypoxia (Figure 2) [29,30,31,32,33]. VHL associates with Elongin-B (*ELOB*) and Elongin-C (*ELOC*), forming a VBC complex, and, together with Cullin-2 (*CUL2*) and E3 ubiquitin-protein ligase RBX1 (*RBX*, Ring-box1), constitutes a functional E3 ubiquitin ligase that specifically recognizes hydroxylated Hypoxia inducible factor (HIF) subunit α, targeting it for proteasome degradation by ubiquitination [34,35,36,37,38,39]. The VHL protein serves as a substrate (e.g., HIF1α) recruitment protein [40,41]. When oxygen levels are normal (normoxic conditions), the two proline residues on the N-terminal (NOOD) and C-terminal (COOD) regions of HIF1α, P402, and P564, respectively, are hydroxylated by the non-heme Fe^2+^ -oxygen and α-ketogluratate-dependent dioxygenase superfamily of prolyl hydroxylases (PHD1, PHD2, and PHD3) [42,43]. In vitro PHD2 catalyzes the hydroxylation of proline in COOD peptides more efficiently than the NOOD peptide proline, whereas PHD3 specifically hydroxylates only COOD proline [44].

The efficiencies of P402 and P564 hydroxylation differ, and when the level of oxygen falls, the hydroxylation of HIF1α prolines is gradually diminished, with P402 more affected than P564 [45,46]. VHL recognizes both hydroxylated prolines with similar binding affinities, resulting in interaction between HIF1α and VBC complex in a 1:2 stoichiometric ratio [47]. HIF1α can therefore bind two VBC complexes or one VBC complex, according to the status of P402 and P564 hydroxylation, at different oxygen levels [45,46,47]. The decrease in VHL targets could lead to gradual increase in HIF1α.

Additionally, in normoxia (normal levels of oxygen), the asparaginyl residue N803 is hydroxylated by factor inhibiting HIF (FIH) or asparaginyl hydroxylase, preventing HIF1α interaction with transcriptional coactivator p300 (*EP300*) [48,49]. Under low levels of oxygen (hypoxia), HIF1α-specific prolyl and asparaginyl residues are not hydroxylated by PHDs and FIH, respectively. Non-hydroxylated HIF1α subunits can enter the nucleus through specific importins, where they form heterodimeric complexes with Aryl Hydrocarbon Receptor Nuclear Translocator (ARNT, also known as Hypoxia-inducible factor 1, β subunit, HIF1β) [43]. The HIF1α-ARNT complexes act as transcriptional regulators, and bind to consensus 5′-(A/G)CGTG-3′ sequence or HIF ancillary sequences 5′-CAGGT-3′ in hypoxia response elements (HRE), within promoters of several genes involved in adaptive response to hypoxia [42,43].

VHL is also involved in numerous HIF-independent pathways, including protein stabilization [50,51], regulation microtubule stability, and ciliogenesis, as well as stability of the mitotic spindle and chromosomal instability [52,53,54], endocytosis [55], apoptosis [56], protein phosphorylation [57], regulation of gene transcription [58,59,60], regulation of extracellular matrix assembly and tight junctions [61,62,63,64], RNA stability [65,66,67], regulation of senescence [68,69], and, in cytokine signaling pathways, mediating DNA damage response and initiating DNA repair [70] or JAK/STAT signaling [71]. These diverse roles of VHL confirm its implication in several crucial cellular processes, including hypoxia-related cellular adaptations, such as hematopoiesis, metabolic adjustments in oxygen deprived environments, and hypoxia-unrelated mechanisms, such as metabolic rewiring, response to oxidative stress, DNA damage, particularly double-stranded breaks, inflammation, and so on, in the absence or presence of reduced oxygen levels.

**Figure 3:**
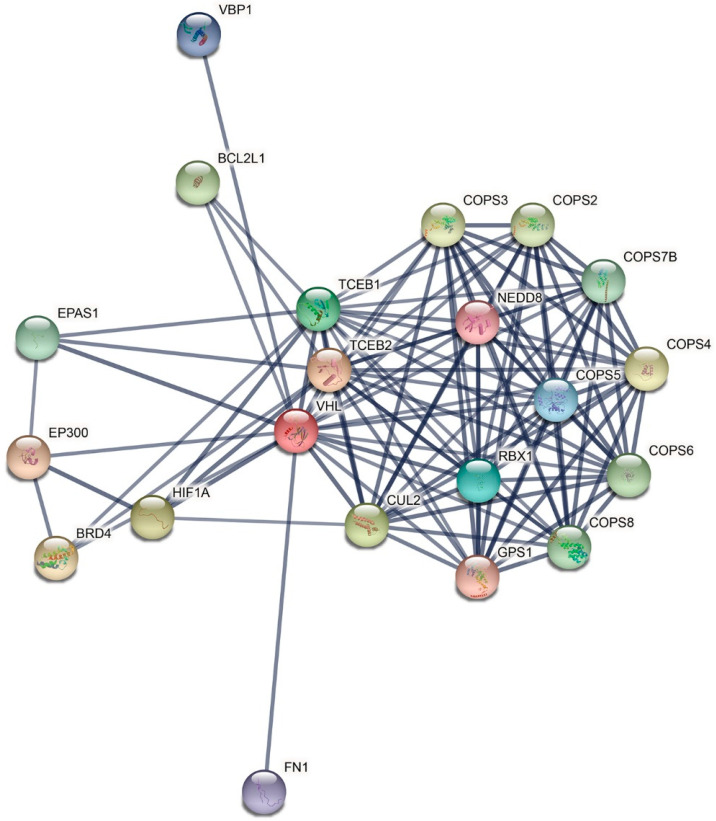
STRING network of VHL protein–protein interactions. Settings: Network type, full STRING network; meaning of network edges, confidence (line thickness indicates the strength of data support); active interaction sources, experiments; minimum required interaction score, high confidence (0.700); max number of interactors to show, no more than 20 interactors; organism, *Homo sapiens* [78].

Due to its involvement in numerous cellular processes, VHL interacts with several proteins, constituting more or less complex molecular relationships (Figure 3). The occurrence of several VHL isoforms further augments the complexity of VHL biological functions. For example, it has been determined that VHL30, but not VHL19, interacts with tumor suppressor ARF (*CDKN2A* gene) through the acidic disordered N-terminus. VHL19 lacks the first 53 N-terminal amino acids in this region, and does not interact with ARF [72,73]. ARF promotes interaction between VHL30 and PRMT3, an arginine methyltransferase which can monomethylate or asymmetrically dimethylate several proteins, including p53 and 40S ribosomal protein RPS2 [72]. Methylated p53 arginine residues affect p53 response by influencing the specificity of p53 binding to promoters [74]. Furthermore, VHL isoforms directly interact with p53, which competes for binding to the VHL α region with Elongin-C and ATM, an important cell cycle checkpoint kinase, as well as AURKA, another cell cycle kinase, which is involved in microtubule formation and the regulation of centrosomes, spindles and kinetochores, and centrosomal proteins, e.g., CEP86 [75,76,77]. VHL isoforms, particularly VHL19, have been shown to interact with collagen, fibronectin, and enzymes involved in collagen biosynthesis [72].

## 3. Clinical Presentation of VHL Disease

Clinical presentation varies in different families, and even in probands within the same family. It is rare to find all manifestations of the disease in one patient. In familial cases, up to 50% of patients have only one manifestation of the syndrome [79]. Clinical criteria for the diagnosis of VHL disease are positive family history for hemangioblastomas in the central nervous system (CNS), or retinal hemangioblastomas; renal cell carcinoma (RCC); pheochromocytoma (PCC), or pancreatic tumors or cysts; and some other tumors. The presence of only one manifestation is sufficient for the diagnosis in familial cases. Diagnostic criteria in sporadic cases are the development of two hemangioblastomas in the CNS or retina, or a hemangioblastoma coupled with visceral tumors or cysts [79,80]. VHL disease is associated with high morbidity and mortality, having a median life expectancy of 50 years. The most frequent causes of death are complications of cerebellar hemangioblastomas (53%) and RCC metastases (32%) [79,81].

There are two clinical types of VHL disease based on the occurrence of typical genotypes and frequencies of RCC or PCC occurrence [3,12,33,82]. Patients without PCC are categorized as type 1 VHL disease, whereas patients with PCC are categorized as having type 2 VHL disease. Type 2 is further sub-classified into three subtypes, A, B, and C, based on the presence or absence of hemangioblastomas and RCC. Type 2A and type 2B both include hemangioblastomas, but RCC is present only in type 2B patients. Type 2C, which is rare [9], includes only PCC, with no hemangioblastomas or RCC [1,80]. Some authors classify Chuvash polycythemia as type 3 VHL disease [83].

## 4. Genetic and Molecular Basis of VHL Disease

The spectra of pathogenic variations in VHL disease is diverse, and the associations between genotypes and phenotypes—the development of specific tumors—are not always predictable [84]. VHL databases, such as VHLdb (http://vhldb.bio.unipd.it/, accessed on 23 December 2021) and the UMD-VHL mutations database (www.umd.be/VHL/, accessed on 23 December 2021), have collected detailed information on over 1600 different pathogenic variations [85]. The COSMIC (https://cancer.sanger.ac.uk/cosmic, accessed on 23 December 2021) database lists more than 1800 entries for genetic variation in *VHL* gene [86]. Based on the diverse roles VHL exerts in cellular homeostasis in normoxic and hypoxic conditions, the elucidation of the effect of pathogenic variations at the molecular level is a daunting task. Over the years, the efforts of several research groups enabled greater understanding of the molecular mechanisms underlying different types of pathogenic genetic variants.

The most common VHL disease-associated genetic changes include deletions of exons, in-frame insertions and deletions, truncating point mutations, missense mutations, splice-site mutations, and frameshift insertions and deletions, as well as structural variations and even gene fusions [9,85,87,88]. In general, from the perspective of VHL genetic modifications, type 1 is characterized by exonic deletions and truncating mutations, as well as missense mutations, associated with VHL instability and high HIF activity [1,12,88]. In type 2, regardless of the subtype, missense mutations, which have generally been found to retain partial functionality of VHL protein, are predominantly found [1,88]. Furthermore, patients with nonsense or frameshift mutations are at higher risk for the development of RCC and hemangioblastomas. Clinical investigations demonstrated that, in addition to the type of the *VHL* genetic variant, the disease type is also age dependent, with a penetrance of over 90% by the age of 65 [79].

Studies involving patients with different manifestations of VHL disease, as well as functional studies of genetic aberrations of *VHL,* have clearly indicated that some aberrations segregate with distinct phenotypes (Table 1). For example, Ong et al. demonstrated that the risk of developing PCC was associated with *VHL* mutations that change surface amino acids (termed surface mutations), compared to mutations that change amino acids buried deeper within the structure (termed deep mutations), and/or large deletions and truncating (frameshift and nonsense) mutations [9]. Furthermore, the mean age at diagnosis was lower in PCC patients harboring surface mutations than in those carrying other types of mutations in the *VHL* gene. They also discovered that RCC and retinal hemangioblastoma patients with deleterious mutations were older at the diagnosis of the disease [9]. Hacker et al. determined that p.R167Q and p.D121G type 2B mutations, located in the Elongin-C binding region, probably retained the ability to bind VBC complex proteins Cullin-2 and Elongin-B, as shown in co-immunoprecipitation experiments [89]. Despite the unstable association of proteins that constitute VBC complexes, this research showed that a p.R167Q VHL mutant could ubiquitinylate HIF1α. Buart et al. showed that an R167Q *VHL* mutation leads to molecular changes related to more pronounced cancer cell stemness and tumor plasticity. VHL-R167Q expressing RCCs are associated with a poor survival [90].

It has been shown that the endothelium of VHL patients is functionally compromised and more susceptible to tumor development [119]. Specific VHL mutations have been associated with defective blood vessel formation. Deletion of *VHL* alleles and certain type 2B VHL missense mutations resulted in an increased risk for hemangioblastoma and RCC formation. These mutations cause abnormal, often excessive, blood vessel remodeling, and data from a study performed by Arreola et al. suggested that they have different effects on the nature of vascular changes during the development of retinal vasculature [115]. They analyzed embryonic stem cell-derived blood vessels with *Vhl*^−/−^, *Vhl*^2B^/^2B^, and WT backgrounds in constructed mouse models with genotypes (i) conditional *Vhl*-null genotype, (ii) one wild-type *Vhl* allele and a second mutant *Vhl* allele with a type 2B p.G518A mutation (equivalent to the VHL p.R167Q protein variation in humans), and (iii) mutant *VHL* p.G518A allele and conditional deletion of the wild type *Vhl* allele (mimicking loss of heterozygosity). Non-mutant mouse models were used as controls. The conditional *Vhl*-null mutation resulted in accelerated arterial vessel maturation, whereas the type 2B *Vhl* p.G518A mutation caused an increase in vessel-branching complexity and disrupted Notch and Vegf signaling, also demonstrated by increased Vegfa, Hey2, and Notch3 mRNA levels in enriched endothelial cells *Vhl*^2B^/^2B^ extracted from embryonic stem (ES) cell cultures. In comparison, the expression profiles of Vegfa and Notch pathway components in *Vhl*^−/−^ endothelial cells were different, indicating that aberrant Vegfa and Notch signaling pathways in different genetic backgrounds differ, and thus influence the morphological differences in the development of vasculature. Examination of postnatal mouse retinas, obtained at different postnatal development stages, demonstrated that conditional *Vhl*-null mutation had a profound effect on the reduction of arterial and venous branching in late stages, and very little effect in early stages. Retinal vessels in *Vhl* heterozygous mice, harboring wild type allele and a type 2B *Vhl* mutation, showed increased arterial, but not venous, branching, whereas in conditional *Vhl* homozygous mice, carrying a type 2B *Vhl* mutation and conditional deletion of second *Vhl* allele, both arterial and venous branching were observed. Collectively, these results indicated the differential effect of aberrant Vegfa and Notch signaling linked to *Vhl* missense mutations, or conditional deletion of *Vhl,* on vascular (dys)morphogenesis [115,120]. These findings could lead to the identification of novel treatment targets in VHL disease, characterized by extensive vascularization due to overproduction of VEGF.

Genotype–phenotype correlations in VHL disease suggest that oxygen-dependent HIF regulation by VHL mutant proteins, as well as HIF-independent VHL functions, modulate the risk of tumor development. It has been established that certain mutations in the VHL gene resulted in a state of pseudohypoxia with elevated levels of HIF proteins, and subsequent activation of HIF-dependent genes, which upregulate angiogenesis, increased cell proliferation and shifted metabolism toward glycolysis, the pentose phosphate pathway, and glutamine-dependent fatty acid biosynthesis, whereas other mutations preferentially affected HIF-independent pathways, without inducing pseudohypoxia [12,91,121]. The HIF signaling pathway is most frequently activated by inactivating mutations of the VHL gene [122]. Abnormally elevated transcriptional activities of the HIF1α and HIF2α genes have been shown to increase tumor survival in solid tumors [123]. Increased hemoglobin concentrations can occasionally occur because of tumor (hyper)production of erythropoietin, as observed in hemangioblastomas, RCC, and PCC [33,122,124,125]. Interestingly, however, despite the fact that elevated HIFs in the background of certain *VHL* mutations has been associated with erythrocytosis, this condition is not a common feature of VHL disease.

The complexity of phenotype–genotype associations between VHL aberrations and disease is further demonstrated by research data that *VHL* genetic aberrations follow the so-called continuum model of tumor suppression, which accounts for the zygosity status of genetic change and tissue specificity [112,126]. Indeed, the research performed by Couve et al. indicated that disease phenotype, in the background of specific *VHL* mutations, can be dependent on the gradient of VHL loss of function, and can show an additive effect in the context of double mutants [112]. Their research resolved intriguing family cases who were classified as having type 2B VHL disease, based on the presence of CNS and retinal hemangioblastomas, RCC, PCC, and pancreatic neuroendocrine tumors. Initially, only heterozygous p.R200W change was found. This change, in hetero- or homozygous form, was firmly associated with normal phenotype and/or erythrocytosis (Chuvash polycythemia), respectively [127,128,129]. Subsequent analyses revealed another change, p.R161Q, located in the same allele together with the p.R200W change, in diseased probands. Protein change p.R161Q was previously associated with type 2A VHL disease, with low risk for the development of RCC. However, the presence of both mutations in the same allele abrogated HIF2α binding, whereas a single p.R161Q mutant showed only partially impaired binding, and p.R200W binding to HIF2α was within the normal range [127,128,129]. Therefore, the simultaneous presence of these two changes in the *VHL* gene affected the HIF signaling pathway more profoundly and carriers of double mutations were susceptible to type 2B VHL disease.

## 5. Clinical and Genetic Features of Selected Manifestations of VHL Disease

### 5.1. Hemangioblastomas in the Central Nervous System (CNS)

Hemangioblastomas of the CNS are the most frequent manifestations in VHL disease. They occur in 60 to 80% of cases and are a presenting lesion in about 40% of cases. The average age of diagnosis is 29 years. Multiple tumors are always associated with VHL disease [79,88]. Most commonly, they are found in the cerebellum, brainstem, and spinal cord. These tumors are benign, and can remain latent for many years. However, tumor growth can result in a mass effect, with variable signs and symptoms, depending on the location and size of the tumor. Hemangioblastomas with cysts tend to grow faster and become symptomatic sooner [130,131]. Patients with cerebellar or brainstem tumors usually present with symptoms of increased intracranial pressure. Ataxia is a common sign of a cerebellar tumor. Tumors in the spinal cord may be associated with neurogenic pain, sensory deficits, proprioceptive changes, paraparesis, and medullar hypertonicity. Paraneoplastic erythrocytosis may be present in some patients due to overproduction of erythropoietin [80].

Hwang et al. examined 55 Korean patients with VHL disease and found that a germline p.E70K mutation was the most frequent in hemangioblastomas of the CNS and retina [97]. They speculated that this change could be a hotspot for Korean patients, due to previous observations that mutations in the β domain that diminish HIFs binding were associated with RCC, CNS, and retinal hemangioblastomas [96,97]. In this cohort of Korean patients, it was associated with single or multiple hemangioblastomas, and was absent in patients with PCC, RCC, and pancreatic cancer [97]. Screening of sporadic CNS hemangioblastoma cases, performed by Catapano et al., identified two patients with heterozygous germline mutations, p.P86L and p.R167W [132]. Interestingly, the patient with a p.P86L mutation developed a retinal hemangioblastoma four years after the treatment of their CNS hemangioblastoma, whereas in the case of a patient with a p.R167W change, a VHL genetic analysis uncovered this change in a close relative who did not have any clinical manifestations. However, all patients were enrolled in VHL-disease surveillance protocol. A large study, that included 533 patients with VHL disease, evaluated the correlation between developing VHL-related tumors and the type of *VHL* mutations [133]. The researchers found that truncating and missense mutations in non-Elongin-C binding sites conferred a high risk for the development of CNS hemangioblastomas. Hong et al. performed a mutational analysis on 540 patients from 80 unrelated families [94]. They confirmed that patients carrying the same mutation, regardless of their relation, can develop distinct tumor phenotypes, which can be related to age, ethnic background, or individual molecular background. Their analyses revealed missense mutations (e.g., c.194C>T (p.S65L), c.262T>C (p.W88R), c.269A>T (p.N90I), c.349T>G (p.W117G), c.481C>T (p.R161*), c.486C>G (p.C162W), and c.500G>A (p.R167Q)) that were found at greater frequencies than expected in patients with CNS hemangioblastomas, although it should be noted that other tumors were also observed in the same or different patients within groups [94].

### 5.2. Retinal Hemangioblastomas and Other Ocular Manifestations

Retinal hemangioblastomas are often the first manifestation of VHL disease. The mean age of onset is 25 years, which is the lowest among other clinical features. Incidence rates have been reported to vary from 49% to 85% [134,135,136]. The likelihood of retinal hemangioblastoma development increases with age, with a probability of 80% after the age of 80 [135]. More than half of patients have bilateral lesions, and around one-third of patients have multiple lesions [134,136,137,138,139,140]. Retinal hemangioblastomas range from small capillary abnormalities to larger lesions that have a typical appearance of a reddish nodule with markedly dilated and tortuous afferent and efferent vessels. Larger tumors can lead to retinal edema, hard exudates, and exudative retinal detachment. Fibrotic changes can result in tractional retinal detachment, which may lead to vitreous hemorrhages, the development of neovascular glaucoma, and eventually phthisis bulbi. Visual loss is generally caused by exudation from the tumor or by traction of glial proliferation on the surface of the tumor. Lesions are mostly located in the retinal periphery (85%); less frequently, they are located near or at the optic disc [12,134,136,137,138,139]. A majority of retinal hemangioblastomas grow over time, but some may be stable for a longer periods, or, although rare, can even spontaneously regress [134,141].

Vascular hamartomas located in the superficial retina were described as small, flat, moss fiber-like vascular lesions, without enlarged afferent and efferent vessels [142]. Juxtapapillary fibrovascular membranes and twin vessels have also been described [134,143,144].

Wittstrom et al. tested retinal function in VHL patients with or without retinal hemangioblastomas [145]. Full-field electroretinography showed significantly prolonged implicit times for rod b-wave (originating from Müller cells and bipolar cells) and maximal combined a-wave (originating from photoreceptors) in VHL patients compared to healthy controls. These results suggested widespread retinal disease even in the absence of a retinal hemangioblastoma or other visible retinal lesions. The same study also showed a significant difference in central retinal thickness between VHL patients and controls; VHL patients had thinner central retinas. This retinal thinning could be secondary to an increased apoptosis of retinal cells [145].

Recently, some researchers analyzed retinal microvasculature using non-invasive optical coherence tomography angiography (OCTA) imaging in patients with VHL disease. Lu et al. analyzed images of 67 eyes with a history of VHL disease, and found significant increases in vessel density of both the superficial and deep capillary plexuses in the macula, regardless the presence of a retinal hemangioma [146]. Similarly, Pilotto et al. found macular perfusion impairment in patients with VHL disease, regardless the presence of a retinal hemangioblastoma [147]. In contrast to the study by Lu et al., patients with VHL disease in this study had lower numbers of retinal vessels in the macular area, and the vessels were thinner in diameter compared to healthy controls. These changes were more pronounced in the superficial capillary plexus [147]. Another study by Pilotto et al. showed thinning of the peripapillary retinal nerve fiber layer in VHL patients without a retinal hemangioblastoma [148]. On the other hand, there was no difference in the peripapillary retinal nerve layer thickness between patients with a retinal hemangioblastoma and healthy controls. The researchers hypothesized that the thinning was a consequence of a reduced perfusion of the radial peripapillary capillary plexus, and that a relative increase in the peripapillary retinal nerve fiber layer, in the presence of a retinal hemangioblastoma, may be due to astrocyte proliferation. All OCTA vascular parameters in the peripapillary area were reduced in VHL patients, and there was no difference between eyes with or without a retinal hemangioblastoma [148].

Optic nerve hemangioblastomas in the intraorbital area, or within the intracranial space, have also been found, although they are very rare. For example, 11 cases (5.3%) were found in a series of 406 patients with VHL disease [137]. Overall, approximately 40 cases of optic nerve hemangioblastomas have been reported in the literature [149,150].

Neovascularization of the iris, and corneal neovascularization, are rare, but have been found in patients with VHL disease. In fact, these are complications of a large retinal hemangioblastoma with longstanding exudative or tractional retinal detachment leading to an extensive retinal ischemia. Iris neovascularization may develop into neovascular glaucoma, and corneal neovascularization may lead to corneal perforation. Both complications result in vision loss [135,137,151].

Genotype analysis performed by Dollfus et al. showed that the majority of patients with retinal hemangioblastomas had missense mutations [152]. Nonsense mutations, frameshifts, and splice site mutations were also detected, albeit at lower frequencies. In this study, in the group of patients with no ocular involvement, in addition to missense mutations, nonsense mutations, and frameshifts, they also found large deletions [152]. Their analyses did not show clear correlations between the location of *VHL* mutation and the phenotype. They observed that the individuals with truncated VHL proteins may have a decreased risk of developing multiple retinal hemangioblastomas in comparison with individuals who have missense mutations in the *VHL* gene. Furthermore, the individuals with truncated protein forms who developed retinal hemangioblastoma had lower visual morbidity than patients with other types of VHL mutations. Similar findings were presented in the study performed by Hajjaj et al., in which patients with missense mutations had worse prognoses, developed more aggressive lesions with progression-related complications, and were at higher risk for developing multiple retinal hemangioblastomas when compared to the group of patients who had truncating mutations [138]. The individuals with missense mutations had a greater chance to develop lesions in all four quadrants [138]. In a large study encompassing 406 individuals from 199 families, more than half of examinees were diagnosed with retinal hemangioblastomas, and, with the exception of very young patients, the majority also had accompanying RCC or CNS lesions [137]. The comparison of mutation types revealed that patients with larger deletions, encompassing the whole VHL gene, were less likely to present with ocular manifestations, whereas partial deletions and nonsense mutations, as well as missense mutations, often resulted in the development of a retinal hemangioblastoma. They also described unusual cases of retinal neovascularization mimicking diabetic retinal neovascularization, which is presumably one of the presentations within the spectrum of hemangioblastoma formation. Most of these patients (88%) did not have a typical hemangioblastoma present in the affected eye, but there was a hemangioblastoma present in the fellow eye in 76% of patients. Retinal neovascularization was most often found at the optic disc, with an epiretinal membrane [137]. Mettu et al. focused on 412 patients with VHL disease who had missense mutations in the *VHL* gene [153]. A total of 159 individuals presented with ocular manifestations, defined as history or clinical evidence in at least one eye. The study found that patients with mutations in the α domain of the *VHL* gene had a higher prevalence of retinal hemangioblastomas compared to individuals with missense mutations in the functional β domain [153]. In addition, these individuals also had a higher risk of developing a juxtapapillary retinal hemangioblastoma, whereas patients with mutations in the β domain more frequently developed peripheral lesions. Overall, in all patients with VHL disease, the most commonly mutated codons of the VHL protein were 98 and 167, followed by codons 78, 117, and 161.

Interestingly, in the following years of research, despite some conflicting results regarding the associations between type and/or location of genetic variations and the severity of the disease emerged, the majority of studies confirmed more frequent involvement of missense mutations and partial deletions in the development of ocular lesion in patients with VHL disease, in contrast to patients who had complete deletion of the *VHL* gene [6,137,138,139,145].

### 5.3. Renal Tumors

Benign renal cysts and malignant RCC are present in two-thirds of patients with VHL disease, and RCC is the leading cause of mortality in VHL disease. The mean age of diagnosis of RCC in VHL disease is 39 years. The risk of RCC varies in different subtypes of VHL disease; however, up to 45% of patients develop multiple and bilateral tumors by the age of 60 years [12,88,130].

Cysts are usually multiple, bilateral, and may be precursors of RCCs. RCCs associated with VHL disease are always of the clear-cell subtype. Büscheck et al. found *VHL* variations in virtually all renal tumor subtypes. They studied the prevalence and clinical significance of *VHL* mutations and 3p25 deletions in renal tumor subtypes, and found highly variable ratios of *VHL* mutations and 3p deletions in the different histological subtypes of kidney tumors. Clear-cell RCC showed the highest rates of *VHL* alterations, exceeding 80% of all cases. In contrast, *VHL* alterations did not exceed 30% in the other renal subtypes. These results are important for developing treatment strategies, indicating that anti-VHL treatment strategies should not be only limited to patients with clear-cell RCC [154].

Most cases of RCCs are asymptomatic, but flank pain or hematuria may be present at the time of diagnosis. Renal lesions often grow slowly; therefore, smaller lesions (less than 2 cm) can be clinically followed up. Tumors larger than 4.5 cm are usually associated with metastases [79,80].

It has been firmly established that HIF deregulation is not sufficient in VHL-defective renal carcinogenesis; rather, several additional genetic events are required to cause RCC, including genetic changes in other genes, aberrant epigenetic events, and so on [155,156,157].

Comprehensive molecular characterization of RCC confirmed the association of *VHL* gene mutations and the involvement of epigenetic reprogramming in the development of the disease [121,158]. PBRM1, or protein polybromo-1, which is a subunit of ATP-dependent chromatin-remodeling complexes, and SET2D, a histone methyltransferase that specifically trimethylates lysine 36 of histone H3 (H3K36me3), were among the top mutated genes in RCC. Interestingly, both genes are located on the short arm of chromosome 3, similar to VHL, in the range of 3p-21 to 3p-25. Interactive network analyses identified 25 sub-networks, among which the subnetwork including VHL and interacting proteins was the most frequently mutated, followed by the PBAF SWI/SNF chromatin remodeling complex, with *PBRM1*, *ARID1A,* and *SMARCA4* as the key mutated genes, and the PI3K/AKT/MTOR pathway, where *PIK3CA* and *MTOR* were the most mutated genes [121]. Comparison of gene expression patterns in patients with *VHL* mutations or chromatin regulators *PBRM1*, *BAP1,* and *SETD2* mutations revealed significant differences between the two backgrounds, as well as differences within the backgrounds of distinct chromatin regulators [121].

Another study, in which the researchers sequenced exomes in 79 samples collected from 10 patients, also confirmed the finding that the most mutated genes in RCC were *VHL*, *SET2D*, and *PBRM1*. Notably, their analyses revealed that *VHL,* and in some cases *PRMB1,* mutations probably occur early in tumorigenesis. They identified an increase of C>T transitions in the context of CpG sites, and a decrease of A>G transitions [159]. Using exome and whole-genome sequencing of paired tumor and normal samples, Sato et al. additionally validated that *VHL*, *SET2D,* and *PRBM1* were among the top five mutated genes in RCC [160]. In addition, in approximately 40% of cases, they identified mutations in *ELOC* (Elongin-C, alias TCEB1), which were linked with the loss of chromosome 8, where *ELOC* is located. The mutations in *ELOC* substituted tyrosine at position 79 with either cysteine or serine, in 7 cases, or alanine 100 with proline. Tyr-79 and Ala-100 are essential for Elongin-C binding to VHL protein [160].

### 5.4. Pheochromocytoma/Paraganglioma

Neuroendocrine tumors, pheochromocytomas (PCCs), and paragangliomas (PGLs), collectively marked as PPGLs, develop from chromaffin cells in the adrenal medulla and parasympathetic or sympathetic ganglia, respectively. In most cases, the tumors are benign, although metastatic disease can develop in approximately 25% tumors [161]. More than one-third and up to 50% of PCCs and PGLs occur as part of inherited syndromes, including multiple endocrine neoplasia type 2 (MEN2), neurofibromatosis type 1, VHL disease, hereditary paraganglioma–pheochromocytoma syndrome, Carney triad, and Carney–Stratakis dyad (National Cancer Institute and [162,163]). PCCs arise in up to 20% of patients with VHL disease. They are usually bilateral, and occur early in life [80]. The mean age at diagnosis is 30 years [9,12]. Patients are often asymptomatic [164]. In contrast to sporadic PCC cases, in many patients with VHL disease there is no excessive catecholamine production by the tumor. However, when there is catecholamine overproduction, some, but not all, patients present with symptoms such as palpitations, sweating, and headache. Undiagnosed catecholamine-producing PCC may result in hypertensive crisis, heart failure, and stroke. Approximately 3% of patients with VHL disease develop a malignant tumor with metastasis [79].

In the context of PPGL molecular taxonomy, genetic aberrations in *VHL, EPAS1,* succinate dehydrogenase subunits *SDHA, SDHB, SDHC,* and *SDHD*, and fumarate hydratase (*FH)*, as well as in *PHD1* and *PHD2*, constitute a pseudohypoxia cluster with specific molecular signatures [162,165,166,167,168]. In VHL-mutated PCC tumor samples, Gao et al. found that seven genes, including *CTGF*, *SDCBP*, *CYR61*/*CCN1*, *COL3A1*, *COL1A1*, *COL5A2*, and *SERPINE1* were significantly up-regulated. This molecular signature proved that *VHL* mutation could promote the development of PCC by activating the expression of cell proliferation- and migration-associated genes [169]. The second cluster, the kinase signaling cluster, is characterized by mutated genes such as *RET*, *NF1*, *TMEM127*, *MAX*, *HRAS*, and *KIF1BB,* whereas the WNT signaling cluster involves mutations in *MAML3* and *CSDE1,* and is mostly associated with aggressive metastatic sporadic PCCs and PGLs [165,168]. Approximately 70% of PCCs can be explained by sporadic or germline genetic aberrations in these genes [170]. Interestingly, certain mutations in *VHL* have been related to a high risk of developing PCCs, a low risk of metastasis, and a low risk of developing PGLs [171]. Furthermore, certain germline *VHL* mutations cause familial PCC without other stigmata of VHL disease (Type 2C VHL disease).

PCCs and low risk PGLs are often bilateral and multicentric, and occur in younger patients with VHL disease, in comparison with other PGL/PCC hereditary syndromes [166,172]. In type 2C VHL disease, which is exclusively associated with the development of PCC, large deletions and truncating mutations are rare, whereas missense mutations, enabling residual VHL function, are more common [1,88,166]. Although the molecular classification of PPGLs included *VHL*-mutated tumors in pseudohypoxia cluster, several lines of research indicated that variations in the penetrance of different *VHL* mutations in the development of PCCs within type 2C, and possibly within other VHL 2 type malignancies, might be associated with HIF-independent effects of VHL [1,88]. In particular, the type 2C p.L188V change in the Elongin-C binding domain does not affect its ability to polyubiquitinate HIFs. Type 2C associated changes in the HIF1α binding domain, p.V84L, p.R64P, and p.F119S, also retained the ability to bind HIF1α or had reduced binding ability, respectively, and were able to polyubiquitinate HIF1α [91]. All type 2C mutants did not bind properly to fibronectin, and were able to suppress GLUT1 levels, which indicated that hypoxia-regulated genes were not induced in the context of type 2C typical *VHL* mutations [91]. The loss of the VHL ability to promote fibronectin matrix assembly could be, therefore, important in type 2C development. Further investigation of *VHL* mutations in type 2C VHL disease also revealed that all type 2C mutants failed to induce apoptosis in adrenal chromaffin cells through inability to stabilize p53 protein and maintain its activity, whereas HIF1α and HIF2α protein levels were not affected [170]. Type 2C mutations p.R64P, p.F119S, and p.L188V have also been shown to interfere with regulation of transcription factor AP-1 (*JUN*)-induced apoptosis. VHL, harboring these changes, failed to reduce transcription factor jun-B levels, and failed to polyubiquitinate atypical protein kinase C family members (aPKC) [92]. Increased jun-B levels have been associated with the suppression of apoptosis in an in vitro rat PCC cell line after removal of nerve growth factor (NGF) [92].

On the other hand, investigation of sporadic and familial cases of PCC identified an exon 2-skipping synonymous genetic variant, c.414A>G, p.Pro138Pro (p.P138P), which produced a shorter *VHL* transcript lacking the HIF binding domain [173]. In five families with type 2A or 2C VHL disease, all affected individuals had PCCs, and type 2A patients also presented with hemangioblastomas, but no RCC. Interestingly, the affected patients were predominantly male. Analysis of TCGA pan-cancer set identified another synonymous variant, p.L188L, which also probably influenced exon 2 exclusion [173]. The complex pattern of *VHL* splicing in the milieu of genetic changes in *VHL* exon and intron regions has been previously demonstrated in patients with VHL disease and erythrocytosis [15]. They observed skipping of exon 2 in investigated LCLs (lymphoblastoid cell lines, established from patients) and PCCs from patients carrying a p.P138P heterozygous change with concomitant loss of the wild-type allele, as well as in patients carrying two heterozygous changes in cryptic exon E′ (c.340+617C>G and c.340+648T>C), but with the wild-type *VHL* allele present. The affected family members in this family were diagnosed either with PCC alone, retinal hemangioblastoma alone, or a combination of PCC, RCC and retinal hemangioblastoma, and CNS hemangioblastoma [15]. Quantification of *VHL* mRNA from samples (LCLs and PCCs) obtained from these patients showed a higher prevalence of transcripts lacking exon 2, and a higher prevalence of transcripts containing E′ in comparison with controls; however, transcripts containing all three exons were still present. Immunoblot analysis subsequently showed that the protein isoform containing E1 amino acids was absent, whereas isoforms VHL213, VHL172, and VHL160 were underrepresented. The analyses of expression using puromycin (inhibitor of nonsense-mediated mRNA decay, NMD) confirmed that the presence of a stop codon in retained E1′ could activate mRNA degradation via NMD [15]. Buffet et al. studied the incidence of germline mutations in the cryptic exon (E1′) of *VHL* gene in patients with VHL disease, and in patients with PCC or PGL. They demonstrated that E′ *VHL* variants are rare, but nearly as frequent as the *VHL* variants in exons 1 and 2 in patients with PCC or PGL [174]. Hergovich et al. discovered that VHL—specifically cytosolic VHL30—interacts with microtubules in vivo, and that amino acid residues 95–123 are necessary for microtubule stabilization and binding [98]. Several disease-associated genetic variations have been located in this region. After performing a series of experiments utilizing wild-type VHL30 and VHL30 carrying selected missense genetic changes, the researchers concluded that altered microtubule stabilization due to the investigated missense genetic changes, p.Y98H and p.Y112H, contributed predominantly to the development of VHL hemangioblastoma and PCC (classified as type 2A VHL disease), whereas p.F119S-induced destabilization of microtubules was characteristic of the type 2C PCC phenotype [98]. Interestingly, alternative substitutions at positions 98, 112, and 119 (p.Y98N, p.Y112N, and p.F119L, respectively), which are associated with type 2B VHL disease, did not show impaired binding to microtubules in vitro. This study indicated that certain substitutions at specific positions within the VHL microtubule-binding region could either destabilize or stabilize microtubules; furthermore, it appears that microtubule stabilization, characteristic of certain type 2B VHL variants, could be incompatible with RCC development [98].

## 6. Case Report—Patient with VHL Disease

A thirty-three year old female began to have severe headaches 14 days before presentation. The headaches were mostly located occipitally, and were more severe in the mornings. One day before presentation, the patient noticed transient left hemifacial tingling. Her past medical history and family history were negative. Neurologic examination revealed mild left upper limb ataxia, dysdiadochokinesis, and right extensor plantar response. Mildly impaired vibration sense was found in the lower limbs. No other abnormalities were found. Romberg’s sign was negative.

**Figure 4:**
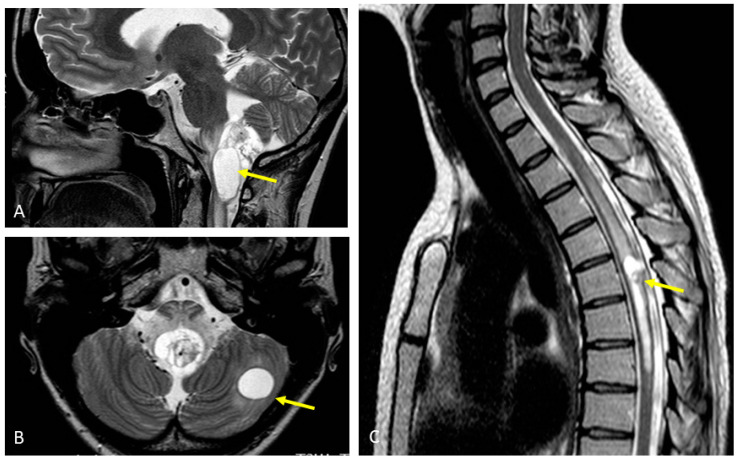
MRI imaging showing CNS lesions of the patient consistent with hemangioblastomas (yellow arrows): (**A**) Lesion pressing the brainstem and upper spinal cord; (**B**) Cerebellar lesion; (**C**) Spinal cord lesion.

The patient underwent extensive diagnostics. Magnetic resonance imaging (MRI) of the head showed a 45 × 20 mm large, partly cystic space-occupying lesion at the base of the 4th ventricle, pressing the brainstem and upper spinal cord (Figure 4A). Two similar lesions were present in the left cerebellar hemisphere (Figure 4B). There were several similar lesions evident in the spinal cord MRI, the largest one being located at the C2 level, and the others at the C5-C6, Th1-Th3, Th5, and Th10 levels (Figure 4C). All lesions were consistent with hemangioblastomas.

**Figure 5:**
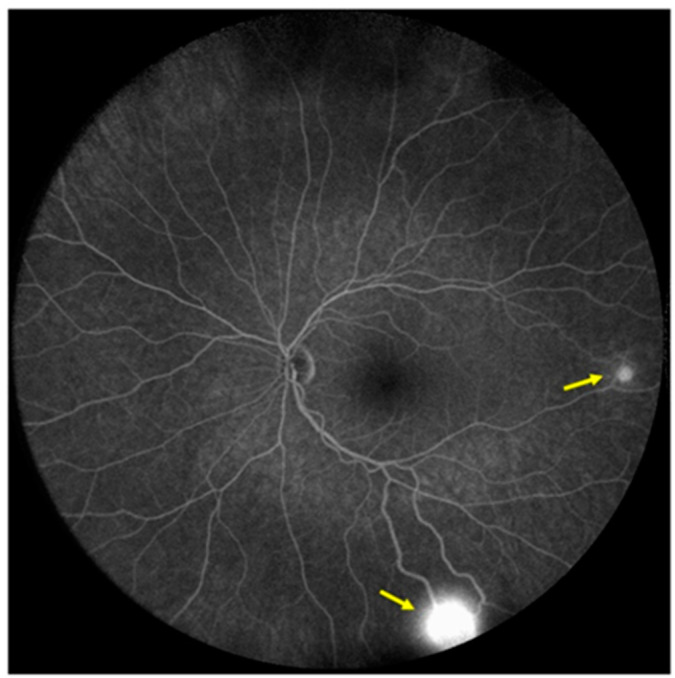
Fluorescein angiography image of the patient’s left eye fundus; yellow arrows show retinal hemangioblastomas.

Two small peripheral retinal hemangioblastomas, some small telangiectatic changes, and a peripheral gliotic lesion with a vitreous traction were found on ophthalmological examination (Figure 5).

Abdominal MRI revealed multiple hepatic and pancreatic cysts and a small cortical cyst in the left kidney. Endocrinological examination ruled out the presence of PCC. The clinical diagnosis of VHL disease was confirmed with genetic testing. A known pathogenic missense mutation, VHL c.194C>T (p.S65L), was found.

Patient first underwent a surgical procedure for the removal of a life-threatening expansive tumor near the 4th ventricle. Histological examination of the excised tumor confirmed the radiological diagnosis of hemangioblastoma (WHO grade I). Brainstem and cerebellar tumors, as well as a spinal cord tumor at the Th5 level, were removed in two additional surgical procedures in the following year. Retinal hemangioblastomas were successfully treated with laser photocoagulation. Since then, the patient has had regular check-ups. In the following three years there was no significant progression of the disease.

### Commentary on the Patient’s Genotype–Phenotype Correlation

Takayanagi et al. identified a c.194C>T transition in VHL-related cerebellum hemangioblastoma, and a nonsense transversion, c.194C>A (p.S65*), in a sporadic case of cerebellum hemangioblastoma, which also harbored additional VHL mutations, c.458T>G and *VHL* promoter methylation, resulting in biallelic inactivation of *VHL* [175]. Using different in vitro approaches, Miller et al. investigated *VHL* codon 65 substitutions p.S65L, p.S65W, and p.S65A [95]. Protein changes p.S65L and p.S65W were previously identified in angiomas and angiomas/hemangioblastomas, respectively [176,177]. All three mutant VHL proteins retained binding capacity for Elongin-B/C, as expected; however, both p.S65L and p.S65W did not bind hydroxylated HIF1α, and were unable to induce HIF2α (EPAS1) degradation. The 65 codon is located in the linker L1 loop, and computational analyses of the substitution of serine with lysine showed that serine plays an important role in the binding of HIF1α to the β domain of VHL, and is possibly also responsible for the dynamic coupling of HIF1α–β domain to the Elongin-C-α domain [95]. A large study, performed on 540 patients, identified a c194C>T mutation, predominantly in CNS hemangioblastomas, followed by RCC, pancreatic tumors/cysts, the genital system (epididymis or broad ligament), retinal hemangioblastoma, and PCC [94].

Determining a genetic cause of the disease presents a good template for establishing optimal clinical management of the patient and genetic counseling for family members.

## 7. Erythrocytosis in the Context of *VHL* Genetic Changes

Erythrocytosis is defined as an abnormally increased red blood cell count [178]. Its occurrence may be seen in various conditions. Interestingly, there are many conditions associated with erythrocytosis, in which erythrocytosis is presumably caused by chronic HIF activation, and yet there are no tumors present in these conditions. Erythrocytosis has been associated with cardiovascular morbidity [179]. According to the retrospective analysis performed by Nguyen at al., the rates of thromboembolic events prior to diagnosis were comparable in both polycythemia vera and secondary erythrocytosis patients. Three out of one hundred and two patients experienced venous thrombosis; all three of these patients were diagnosed with polycythemia vera [180]. Changes in blood composition disturb microvascular circulation and lead to hyperviscosity and thrombotic events. Ophthalmological examination of the fundus may reveal venous dilatation and tortuosity, intraretinal hemorrhages, retinal edema, hard exudates, cotton wool spots, and optic disc edema. Although ocular changes have been well documented in patients with polycythemia vera [181,182,183,184], and may occur early in the progression of the disease, the occurrence of ocular lesions in other types of erythrocytosis is less well documented.

It has been established that the inheritance of *VHL* genetic changes is an important cause of secondary erythrocytosis [185]. Up to 50% of patients with apparent congenital erythrocytosis and elevated serum EPO appear to have a mutation in the *VHL* gene [186]. The OMIM (https://omim.org/, accessed on 24 December 2021) database categorizes *VHL*-induced erythrocytosis as familial erythrocytosis ECYT2, displaying features of both primary and secondary erythrocytosis, such as hypersensitivity of erythroid progenitors to circulating EPO, as well as raised blood EPO levels [187].

Chuvash polycythemia, first observed in the Russian Chuvash population and associated with a p.R200W change in the *VHL* gene [127,128,129], was later found in patients of other ethnic groups [124,186,188]. Subsequent genetic analyses revealed several other missense homozygous and compound heterozygous genetic changes in *VHL* as the underlying cause of this disease [186]. Gordeuk et al. analyzed vascular complications among patients with Chuvash polycythemia and found a positive history of major arterial or venous thrombosis in 24% of patients, compared to only 4% of controls. In addition, a history of bleeding was found in 32% of patients with Chuvash polycythemia, compared to 4% of controls. An increased risk for thrombosis was associated with homozygosity for the 598C>T (p.R200W) *VHL* change. Interestingly, patients with Chuvash polycythemia had no CNS hemangioblastomas, RCCs, or PCCs [124,189]. It has been established that quantitative differences with respect to HIF, and qualitative differences with respect to HIF-independent VHL functions, probably account for the low renal cancer risk associated with Chuvash polycythemia and the high renal carcinoma risk associated with complete loss of function of VHL in renal cells [112,125]. In addition to ECYT2, caused by homozygous (or compound heterozygous) hypomorphic *VHL* alleles, secondary erythrocytosis can result from genetic changes in other genes in the oxygen-sensing pathway, such as *EPAS1* (HIF2α protein) alleles (ECYT4) or *EGLN1* (PHD2 protein) alleles (ECYT3). This suggests that subtle defects in the VHL-PHD2-HIF2α pathway can cause polycythemia without significant increase of carcinogenesis [125,190].

Pastore et al. suggested there is a distinction between “polycythemia-causing” VHL variations and other *VHL* variations causing VHL syndrome with tumor development [186]. They investigated patients with polycythemia from different ethnic backgrounds and found homozygous and compound heterozygous changes, including 598C>T (p.R200W), 571C>G (p.H191D), 562C>G (p.L188V), and 574C>T (p.P192A). They concluded that compound heterozygous changes, such as P192A and L188V in one allele and “polycythemia-causing” p.R200W in the second allele, could be termed “polycythemia associated” *VHL* genetic changes. These changes are located in the C-terminal region, at the end of the Elongin-C binding region, or just outside it. It has been shown that L188V affected VHL–fibronectin interaction in the context of RCC and PCC, and did not impair HIF1A degradation [91,186]. They speculated that it is possible that the newly identified changes in *VHL* also do not greatly affect VHL function in HIF1α regulation [186]. In a functional investigation of p.R200W change, the researchers identified enhanced p.R200W and p.H191D VHL interaction with SOCS1, which interfered with JAK2 recruitment and subsequent degradation of JAK2 [71]. The resulting JAK2 stabilization and JAK2-STAT5 stimulation of erythroid progenitors in these patients could, in part, explain hypersensitivity to EPO, which is characteristic of primary erythrocytosis [71]. These findings could be exploited further to investigate whether patients with Chuvash polycythemia would respond to pharmacologic JAK2 inhibition.

However, further research of the homozygous change p.H191D, which was present in one male patient with elevated EPO from Croatia [186], and subsequently confirmed in another Croatian female patient with elevated EPO [191], uncovered the possibility that different mechanisms could drive pathogenic characteristics in carriers of p.R200W (Chuvash) and p.H191D (Croatian) variants. The authors observed that EPO concentrations in both patients with the p.H191D variant were higher than in carriers of a homozygous p.R200W change. Native erythroid progenitors of p.H191D carriers were not hypersensitive to EPO, indicating that this phenotype could be driven exclusively by EPO [191].

Next, despite initial observations that Chuvash polycythemia and congenital erythrocytosis *VHL* genetic changes clustered in exon 3, it has been found that different changes in exon 2 could also underlie the development of erythrocytosis, without malignant manifestations characteristic of *VHL* changes in other exons. For example, the discovery and investigation of another homozygous variant, p.D126N in exon 2, in one pediatric patient with early onset pulmonary hypertension, polycythemia, and multiple hepatic hemangiomas, also indicated only mild EPO hypersensitivity of erythroid progenitors. Expression analyses showed normal levels of *NF-E2* and *RUNX1* mRNA, which are associated with EPO hypersensitivity in primary and Chuvash (p.R200W) polycythemias [192,193]. This patient had remarkably high EPO concentrations—higher than those present in patients with Chuvash (p.R200W) or Croatian (p.H191D) variants [193].

Lanikova et al. identified a homozygous exon 2 VHL P138L variant in a patient with congenital polycythemia [194]. Heterozygous relatives did not present any cancerous or non-cancerous manifestations of VHL disease. Functional analyses showed an association between p.P138L and an increase in the expression of HIF-regulated genes *SLC2A1*, *TFRC*, and *HK*, as well as *RUNX1/AML1* and *NF-E2*, which are increased in acquired and primary polycythemia, respectively. The mutated VHL protein also showed reduced stability and decreased ability to polyubiquitinate HIF1α [194]. Lenglet et al. used several approaches to discern the functional effects of *VHL* genetic changes in patients with erythrocytosis and VHL disease [15]. They demonstrated that synonymous heterozygous mutation in patients with erythrocytosis induced the expression of an additional VHL isoform, a 193 amino acid protein containing the first 114 amino acids of exon 1 and an additional 79 amino acids spliced from cryptic exon E1′. They also identified polymorphic sites in E′ (c.340+1770T>C, c.340+1694_711dup, and c.340+1816A>C) in patients with erythrocytosis, constituting complex compound genotypes in these patients. Similar to the previously mentioned observation in PCC patients with distinct *VHL* variations, expression studies of these variations also indicated that, despite higher mRNA levels of transcripts containing E′, they could not detect E′-containing VHL isoforms, indicating that these transcripts may be targeted for NMD. A synonymous homozygous p.D143D change in patients with erythrocytosis was associated with exon 2 skipping, resulting in elevated levels of transcripts containing only exons 1 and 3; however, western blot analysis showed that all VHL isoforms were downregulated. Further analysis of variants, associated with erythrocytosis, p.G144R, and p.P138L, as well as a variant, p.P138P, which was associated with VHL disease, revealed that their effect on splicing is differential, with most splicing events occurring in p.P138P background [15].

In another study, investigation of cases and relatives with unexplained erythrocytosis revealed that 61 of 84 patients with suspected congenital erythrocytosis were carriers of an intron genetic variant, c.-195G>A [195]. Further screening of 78 probands and their relatives indicated that AA homozygotes and GA heterozygotes were significantly overrepresented in patients’ group. This research indicates that idiopathic erythrocytosis could be explained in the background of common single genetic variations (SNVs), possibly in interaction with other SNVs in *VHL* or VHL binding partners.

## 8. Conclusions

Hereditary genetic changes in the *VHL* gene that result in the development of different tumors and erythrocytosis show great complexity, and present a significant challenge with regard to the elucidation of variants association with phenotypes. Recent research has revealed the involvement of VHL in different cellular processes and molecular pathways, both HIF-dependent and HIF-independent, as well as its ability to interact with many different proteins. Therefore, it is expected that the pathogenic effects of *VHL* variants will influence different mechanisms in cells. The association of VHL variants located at different positions, and the association of different amino acid substitutions at the same position, with distinct phenotypes, could, in the future, aid in the uncovering of pathogenic mechanisms, leading to the development of particular phenotypes of the disease. The identification of VHL-interacting protein partners could further shed light on molecular pathways involved in the initiation and progression of VHL disease. This, in turn, could lead to the development of novel therapeutic approaches and the possibility of optimizing follow-up procedures for carriers of VHL mutations. In addition, the finding that synonymous genetic changes of *VHL* can influence mRNA splicing mechanisms is important, not only for elucidation of VHL disease mechanisms, but also for reconsideration of synonymous changes and their possible deleterious effects on mRNA maturation in other pathological conditions.

Nevertheless, besides family history and clinical presentation, molecular genetic testing of *VHL* variations is becoming an important tool in confirming the diagnosis. Genetic testing proved to be indispensable in patients with unclear family history. Understanding the pathogenic effects of *VHL* variants might better predict the prognosis and help medical professionals to optimize the management of the patient.

## Figures and Tables

**Table 1:** Selected VHL genetic variants in VHL disease and their association with phenotype.

Variant	Protein Change	Codon	VHL Type/Phenotype	Functional Consequence	Reference
c.191G>C	R64P	64	Type 2C	Increased aPKC JUNB levels; impaired binding to fibronectin.	[91,92]
c.194C>T	S65L	56	Type 2B	Impaired HIF1α binding; impaired HIF2α regulation.	[93,94,95]
c.208G>A	E70K	70	Type 1	Impaired HIF1α binding.	[96,97]
c.233A>G	N78S	78	Type 1	Impaired HIF1α regulation.	[98,99]
c.239G>A	S80N	80	Type 2C	No known consequence.	[98]
c.245G>C	R82P	82	Type 2B	Loss of function of VHL.	[100]
c.250G>C	V84L	84	Type 2C	No known consequence.	[98]
c.262T>A c.262T>C	W88R	88	Hemangio- Blastoma ^1^	No known consequence.	[94]
c.269A>T	N90I	90	Type 2B	Impaired HIF1α regulation.	[94,98,101,102]
c.292T>C	Y98H	98	Type 2A	Impaired HIF1α regulation; defective microtubule stabilization.	[91,98]
c.292T>A	Y98N	98	Type 2B	Impaired HIF1α regulation; impaired GLUT1 suppression.	[101]
c.334T>A	Y112H	112	Type 2A	Impaired HIF1α regulation; decreased VHL stability.	[89,103]
c.334T>A	Y112N	112	Type 2B	Reduced stability of the Vhl-Elongin B/C complex; impaired HIF1α regulation; elevated HIF2α, GLUT1, and cyclin D1 expression in normoxic conditions.	[103,104,105]
c.334T>G	Y112D	112	Type 2C	No known consequence.	[98]
c.340G>C	G114R	114	Type 2B	Reduced stability of the Vhl-Elongin B/C complex.	[106]
c.349T>C c.349T>A	W117R	117	Type 2B	Impaired HIF1α regulation; impaired binding to fibronectin; elevated HIF2α and GLUT1 expression in normoxic conditions.	[62,105,107]
c.355T>C c.357C>G c.357C>A	F119L	119	Type 2B	Decreased VHL stability; impaired HIF1α regulation.	[108]
c.407T>C	F136S	136	Type 2B	No known consequence.	[98]
c.407T>A	F136Y	136	Type 2B	No known consequence.	[98]
c.408T>G	F136L	136	Type 2B	Decreased VHL stability; impaired HIF1α regulation.	[108,109]
c.482G>C	R161P	161	Type 2B	Reduced stability of the Vhl-Elongin B/C complex; defective microtubule stabilization.	[110,111]
c.482G>A	R161Q	161	Type 2A; Type 2B	Reduced VHL stability.	[112]
c.486C>G	C162W	162	Hemangio- Blastoma ^1^	Impaired HIF1α regulation.	[94,113]
c.499C>T	R167W	167	Type 2B	Decreased binding to Elongin B/C and Cullin-2; impaired ubiquitination and degradation of ESR1.	[62,114]
c.500G>A	R167Q	167	Hemangio- Blastoma ^1^	Decreased binding to Elongin C; impaired HIF2α regulation.	[94,115,116]
c.562C>G	L188V		Type 2C	Impaired binding to fibronectin; elevated RWWD3, aPKC, and JUNB levels.	[91,92,117,118]

^1^ type of VHL disease not classified.

## Data Availability

In preparation of the manuscript, the following publicly available datasets were used: Human Protein Atlas, version 20.1 (https://www.proteinatlas.org/) (accessed on 10 January 2022), STRING (https://string-db.org/) (accessed on 14 January 2022), VHLdb (http://vhldb.bio.unipd.it/) (accessed on 23 December 2021), UMD- VHL mutations database (www.umd.be/VHL/) (accessed on 23 December 2021), COSMIC (https://cancer.sanger.ac.uk/cosmic) (accessed on 23 December 2021), NCBI RefSeq (https://www.ncbi.nlm.nih.gov/refseq/) (accessed on 24 December 2021), cBioPortal (https://www.cbioportal.org/) (accessed on 30 December 2021).
